# Serological Evidence of Backyard Pig Exposure to Highly Pathogenic Avian Influenza H5N8 Virus during 2016–2017 Epizootic in France

**DOI:** 10.3390/pathogens10050621

**Published:** 2021-05-18

**Authors:** Séverine Hervé, Audrey Schmitz, François-Xavier Briand, Stéphane Gorin, Stéphane Quéguiner, Éric Niqueux, Frédéric Paboeuf, Axelle Scoizec, Sophie Le Bouquin-Leneveu, Nicolas Eterradossi, Gaëlle Simon

**Affiliations:** 1 Swine Virology Immunology Unit, National Reference Laboratory for Swine Influenza, Ploufragan-Plouzané-Niort Laboratory, French Agency for Food, Environmental and Occupational Health and Safety (ANSES), 22440 Ploufragan, France; stephane.gorin@anses.fr (S.G.); stephane.queguiner@anses.fr (S.Q.); gaelle.simon@anses.fr (G.S.); 2 Avian and Rabbit Virology Immunology and Parasitology Unit, National Reference Laboratory for Avian Influenza, Ploufragan-Plouzané-Niort Laboratory, French Agency for Food, Environmental and Occupational Health and Safety (ANSES), 22440 Ploufragan, France; audrey.schmitz@anses.fr (A.S.); francois-xavier.briand@anses.fr (F.-X.B.); eric.niqueux@anses.fr (É.N.); nicolas.eterradossi@anses.fr (N.E.); 3 SPF Pig Production and Experimentation, Ploufragan-Plouzané-Niort Laboratory, French Agency for food, Environmental and Occupational Health and Safety (ANSES), 22440 Ploufragan, France; frederic.paboeuf@anses.fr; 4 Epidemiology, Health and Welfare Unit, Ploufragan-Plouzané-Niort Laboratory, French Agency for Food, Environmental and Occupational Health and Safety (ANSES), 22440 Ploufragan, France; axelle.scoizec@anses.fr (A.S.); sophie.lebouquin-leneveu@anses.fr (S.L.B.-L.)

**Keywords:** poultry, swine, influenza outbreak, mixed herd, hemagglutination inhibition test

## Abstract

In autumn/winter 2016–2017, HPAI-H5N8 viruses belonging to the A/goose/Guandong/1/1996 (Gs/Gd) lineage, clade 2.3.4.4b, were responsible for outbreaks in domestic poultry in Europe, and veterinarians were requested to reinforce surveillance of pigs bred in HPAI-H5Nx confirmed mixed herds. In this context, ten pig herds were visited in southwestern France from December 2016 to May 2017 and serological analyses for influenza A virus (IAV) infections were carried out by ELISA and hemagglutination inhibition assays. In one herd, one backyard pig was shown to have produced antibodies directed against a virus bearing a H5 from clade 2.3.4.4b, suggesting it would have been infected naturally after close contact with HPAI-H5N8 contaminated domestic ducks. Whereas pigs and other mammals, including humans, may have limited sensitivity to HPAI-H5 clade 2.3.4.4b, this information recalls the importance of implementing appropriate biosecurity measures in pig and poultry farms to avoid IAV interspecies transmission, a prerequisite for co-infections and subsequent emergence of new viral genotypes whose impact on both animal and human health cannot be predicted.

## 1. Introduction

Influenza A viruses (IAVs) infect a wide range of animal hosts, sometimes crossing the species barrier. Swine IAVs (swIAVs) originate in toto from human or avian species or are reassortant viruses with genomic segments of different origins, obtained from swine adapted, human or avian IAVs. Thus, multiple genetic lineages exist among enzootic H1N1, H1N2 and H3N2 swIAV subtypes, depending on geographical areas and local histories of successive reassortment events [1]. IAVs of other subtypes, such as H3N1, H1N7, H4N6, H3N3, H9N2, H2N3, H5N1, H7N2, H6N6, H4N1, H5N2 and H5N6 [2,3,4,5,6,7], have been described in pigs sporadically, but no evidence has been found for their adaptation. Most of these examples were IAVs of avian origin, but to date the so-called “Eurasian avian-like swine H1N1” (H1_av_N1) virus that emerged in Europe in 1979 and reached Asia in 2000’s is the only avian IAV that became enzootic in pigs without further reassortment with a previously adapted swIAV [8,9]. In Europe, this enzootic swine H1_av_N1 virus further reassorted with human H3N2 and H1N1 viruses, leading to the co-circulation of reassortant swIAVs of H3N2 subtype from 1984 and H1_hu_N2 subtype from 1994, respectively [2]. After the 2009 influenza pandemic due to a swine-origin IAV, the H1N1 pandemic virus (H1N1pdm) transmitted back from humans to pigs worldwide. In Europe, the three previous enzootic swIAVs underwent reassortments with each other, with H1N1pdm or with seasonal human H3N2 virus, but none of the swIAVs identified in European pigs in the last years exhibited genes from avian viruses that would have been transmitted from birds to pigs recently [10,11,12,13,14,15]. Challenge studies confirmed that pigs could be infected with avian IAVs, but in most cases they did not induce clinical signs. They only replicated in the lower lung in consistence with the relative distribution of avian IAV receptors (alpha-2,3 sialic acids) in pig respiratory tract, and transmitted poorly or even not at all between pigs or from pigs to other mammals [15,16,17,18,19]. However, pig sensitivity to avian IAVs may depend on various factors such as virus subtype, infectious dose and/or route of inoculation [15]. Thus, questions remain about the risk that avian IAVs are transmitted to pigs in case of high infection pressure and/or close contacts between species, especially when highly pathogenic avian influenza (HPAI) viruses are responsible for severe outbreaks in mixed pig–poultry farming. European member states, including France, are vigilant on this regard and recommend monitoring of pigs in such situations [20,21].

In autumn/winter 2016–2017, HPAI-H5N8 viruses from A/goose/Guandong/1/1996 (Gs/Gd) lineage, clade 2.3.4.4b, were responsible for outbreaks in domestic poultry in Europe [22,23,24,25,26,27]. Death rate was lower than for previous 2005–2006 and 2014–2015 HPAI-H5 epizootics, but the magnitude of the 2016–2017 epizootic, considering the numbers of affected farms, and countries and the diversity of infected wild bird species, was unprecedented with a huge economic impact [23]. In France, 538 cases, i.e., 52 wild birds and 486 poultry flocks, of HPAI-H5N8 infections were reported from November 2016 to June 2017 [23,25,26]. The duck production sector located in the southwestern part of the country was the most affected, first with local farm-to-farm spreading of the disease, then with an increased average distance between affected farms as it extended westwards. The veterinarians were requested to reinforce clinical follow-up of pigs bred in HPAI-H5N8 confirmed mixed herds and to carry out serological surveys to know if the virus had crossed the species barrier and had contaminated pigs.

## 2. Materials and Methods

### 2.1. Herds and Sampling

**Figure 1:**
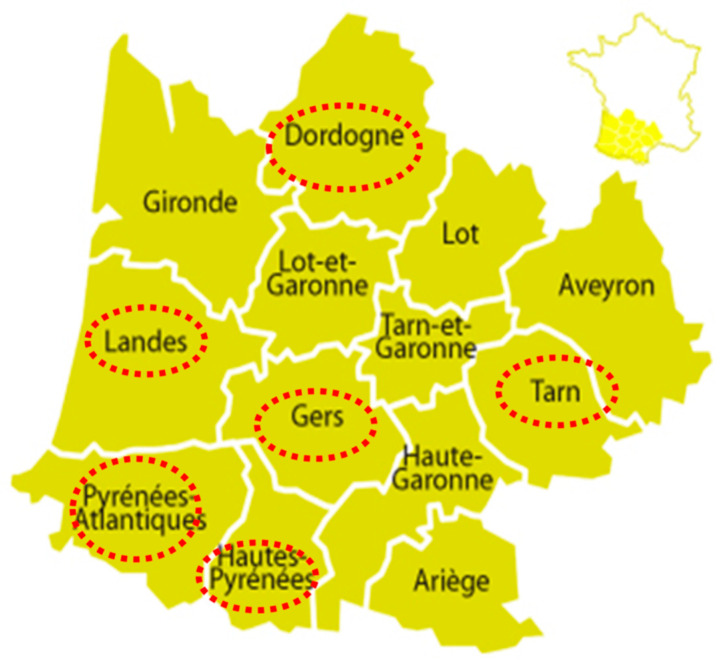
Localization of pig herds sampled in the frame of the 2016–2017 HPAI-H5N8 epizootic in southwestern France. Names of concerned administrative regions (départements) are circled in red.

Ten pig herds (identified #1 to #10) located in six départements from the southwestern part of the country were visited by veterinarians from December 2016 to May 2017 (Figure 1 and Table 1). They were all pig–poultry mixed farms, except herd #1, with variable swine production types and sizes. Whereas herd #1 did not meet the inclusion criteria, it was still kept in the study as it was an outdoor farm that may have been exposed to avian viruses from wildlife.

Sows and growing pigs were sampled for serological investigations. Blood was taken once in herds #1, #5, #6, #7, #8 and #10, twice approximately four weeks apart in herds #3, #4 and #9, and on three occasions in herd #2. The numbers of sampled animals varied from 1 to 60 per visit, depending on the production type.

### 2.2. ELISA

Sera were tested by enzyme-linked immunosorbent assay (ELISA) for detection of antibodies directed against the IAV nucleoprotein, using ID Screen^®^ Influenza A Antibody Competition Multi Species (IDVet, Grabels, France) according to manufacturer’s instructions [28,29]. The specificity of the test is 100% (CI_95_: 97.7–100%) with a CV comprised between 6% and 9% for the repeatability. A herd was considered positive when at least one serum tested positive.

### 2.3. Virus Strains Used in Hemagglutination Inhibition Tests

ELISA positive batches were submitted to hemagglutination inhibition (HI) tests to assess antibodies directed against the IAV hemagglutinin (HA). Sera were tested against antigens representative of swIAVs enzootic in pigs throughout Europe currently [14,29] and used for routine serological diagnostic in pigs in France at the time of the survey (Table 2). Avian antigens were included in HI tests in order to detect and discriminate anti-H5 antibodies in the context of the 2016–2017 epizootic (Table 2). 

Two antigens were HPAI-H5 viruses belonging to clade 2.3.4.4b, i.e., A/decoyduck/France/161105a/2016, representative of H5N8 viruses circulating in France during the 2016–2017 epizootic, and A/muteswan/Croatia/102/2016, a H5N5 strain representative of other HPAI-H5 viruses circulating in Europe at the same time. Two others were H5 strains belonging to lineages significantly different to the Gs/Gd lineage: A/duck/France/150236b/2015, an HPAI-H5N9 strain isolated during the 2015–2016 epizootic, and A/muscovyduck/France/070090b/2007, a H5N3 strain representative of European H5 low pathogenic avian IAVs (LPAI). Finally, two HxN8 viruses, A/mallard/France/100204f/2010 (H3N8) and A/pekinduck/France/090173/2009 (H6N8), were included to evaluate any potential cross-reaction of the N8 neuraminidase in the HI test as recommended by the European commission for surveillance of avian influenza [30,31]. Moreover, human strain A/Victoria/361/2011 (kindly provided by M. Rosa-Calatrava, VirPath Laboratory, Lyon, France) was included as a representative of seasonal H3N2 viruses that were circulating during the 2016–2017 or previous epidemics in humans.

### 2.4. Production of Negative and Positive Control Sera in Specific Pathogen Free Pigs

Negative and positive pig sera were included as controls to ensure accurate HI tests for the detection of anti-H5 antibodies in swine and assess potential cross-reactions. Negative sera were obtained from specific pathogen free (SPF) pigs, bred in ANSES biosecurity level 3 facilities, Ploufragan, France. Hyper-immune sera (HIS) directed against the reference swIAVs were previously produced in SPF pigs, involving intranasal inoculation of live virus followed by intramuscular injection of live virus in the presence of adjuvant [32]. HIS directed against avian IAVs were specifically produced in this study. This type of experiment was approved by the French national committee for ethics in animal experimentation ANSES/ENVA/UPEC (approval n°12/07/16-1) and authorized by the French Ministry of Research (APAFIS #2016060715424878 v2). Briefly, the H5N8, H5N9 and H5N3 strains were propagated in 11-day-old SPF embryonated chicken eggs and inactivated for 3 h at 37 °C in 0.05% beta-propiolactone (*v/v*). Each inactivated antigen was inoculated to one 9-week-old SPF pig. Three mL of inoculum, composed of inactivated strain and Montanide ISA 206 adjuvant (Seppic, La Garenne-Colombes, France) mixed in a 1:1 ratio, were injected intramuscularly at three occasions, at days 0, 21 and 35. Blood samples were taken weekly until day 49 when pigs were euthanized and HIS were collected.

### 2.5. Hemagglutination Inhibition Tests

Sera obtained from pigs sampled in investigated herds as well as reference HIS were treated to inactivate nonspecific hemagglutination inhibitors and to remove non-specific agglutinins as described in standard protocol [33]. Briefly, 4 volumes of receptor-destroying enzyme (RDE) were added to 1 volume of serum. The mixture was incubated overnight at 37 °C, followed by incubation at 56 °C with 5 volumes of 1.5% sodium citrate solution for 30 min. Finally, chicken erythrocytes, previously washed twice with PBS (pH = 7.2) before suspension at 50% in PBS, were added in 1/10 volume of the serum and incubated at 4 °C under gentle shaking for 1.5 h, before removing erythrocytes by centrifugation at 1000 *g* for 10 min at 4 °C. HI tests were performed using 4 hemagglutinating units of virus with 0.5% chicken erythrocytes according to standard procedures [29,33]. Two-fold treated serum dilutions were tested starting from 1:10 dilution.

## 3. Results

No clinical signs were reported in any of the ten sampled herds.

Herds #1, #2 and #9 were detected IAV seropositive in ELISA (Table 1). In non-mixed herd #1, the ten sows sampled in January 2017 were positive. In farm #2 there were only two backyard pigs. One serum out of two taken late December 2016, 22 days after the H5N8 infection was confirmed in ducks, was found positive, as well as the two sera taken six weeks later in February 2017 and the serum obtained in May 2017. In herd #9, a batch (A) of growing pigs sampled at 10 weeks of age and three weeks later tested positive, with 43% and 30% positive animals, respectively. Some animals were positive at both visits (data not shown). These pigs were born in a farrowing distinct site and integrated the post-weaning-finishing herd after the H5N8 outbreak started in ducks. Thirty finishing pigs from another batch (B) were sampled at 17 and 20 weeks of age, respectively, but they all tested negative. In other mixed herds (#3, #4, #5, #6, #7, #8, #10), all sampled pigs tested negative (Table 1).

Before analyzing the ELISA positive sera in HI tests using the panel of selected swine, avian and human IAVs, cross-reactions were evaluated by testing these antigens with the HIS produced in SPF pigs. Negative sera from non-inoculated SPF pigs did not react (HI titer <10) with any antigen (data not shown). Thus, the positivity threshold of HI tests has been set at HI titer of 20, titer of 10 being considered as non-significant. Homologous HI titers of anti-swIAV HIS ranged from 640 to 2560 and some cross reactions occurred between some swine subtypes (Table 2), as usually measured [32]. The pigs inoculated with inactivated avian IAVs in the presence of adjuvant also produced specific antibodies that were detected from one week after the first boost (data not shown). Titers further increased slightly after the second boost (third injection) even if HIS against inactivated avian strains did not reach the same levels as HIS produced after immunization with live swIAV (Table 2), probably because avian strains were inactivated before intramuscular inoculation while first immunization with swIAVs was operated using live strains via the nasal route. Incidentally, HIS obtained against H5N8, H5N9 and H5N3 strains were positive, exhibiting mean HI titers of 50, 25 and 160 in homologous reactions, respectively (Table 2). Anti-H5N8 HIS also reacted with H5N5 antigen, in line with the fact that both H5 belong to the same Gs/Gd lineage, clade 2.3.4.4b, and in accordance with Kaplan et al. who described such a cross-reactivity among avian H5Nx clade 2.3.4.4 strains in experimentally infected pigs [18]. However, it did not present any cross-reaction with other H5Nx or HxN8 antigens. By contrast, cross-reactions were observed between H5N9 and H5N3 strains, the strongest reaction occurring between anti-H5N3 HIS and the H5N9 antigen. No positive reactions were evidenced either between anti-H5 HIS and swIAVs or human H3N2, or between anti-swIAV HIS and H5Nx antigens or human H3N2 (Table 2). Altogether, these data showed that the HIS produced in SPF pigs against H5N8, H5N9 and H5N3 strains, might serve as reliable controls for the interpretation of HI tests including avian antigens with the aim to detect antibodies directed against H5N8 clade 2.3.4.4b in pigs.

Armed with these data, we then analyzed in HI tests the field sera taken in herds previously found IAV seropositive in ELISA. In non-mixed herd #1, 7/10 sow sera exhibited HI titers ranging from 20 to 160 towards both H1_av_N1 and H1N1pdm swIAVs but none of them showed any positive titer towards avian and human antigens (Table 3). Even if some cross-reaction exists between H1_av_ and H1pdm, both viruses were assumed having circulated in this farm based on individual HI titers, which is consistent with data from the swIAV surveillance conducted at that time in this region [36]. This is also in accordance with the fact that no HPAI-H5 outbreak was reported in wild birds or poultry farms in this département during the epizootic [26].

In mixed-herd #2, the sera obtained from the two backyard pigs did not exhibit any reaction with the swIAV reference antigens (Table 3). By contrast, the sera taken on pig #1 in December 2016 and February 2017 exhibited HI titers above the positivity threshold with H5N8 and H5N5 antigens, both of them having a H5 from clade 2.3.4.4b. On the contrary, they did not react either with the H5N9 and H5N3 strains with H5 gene that do not belong to the Gs/Gd-lineage, or with two other HxN8 strains (Table 3). Antibodies against avian viruses were not detected in sera from the second backyard pig (pig #2), but this animal was found seropositive towards the human H3N2 antigen, suggesting it had been infected with a seasonal virus. One of the two backyard pigs was slaughtered between the second and the third sampling. The serum taken in May 2017 on the remaining pig gave negative HI results with all antigens, probably because of low levels of anti-IAV antibodies following natural decrease, as suggested by the S/P value obtained in ELISA, which was close to the method threshold for this sample. Thus, analyses conducted in herd #2 led to hypothesize that pig #1 had been most probably infected with an H5Nx virus from clade 2.3.4.4b a few weeks before sampling on 23 December, e.g., possibly the epizootic HPAI-H5N8 responsible for the outbreak that was confirmed in ducks in this farm #2 on 1 December [25,26]. Virus transmission could have happened through direct contact between species and/or exposure to fomites, since ducks and backyard pigs would have shared the same outdoor route on the farm. It could also be linked to the supply of contaminated food, as hypothesized in mixed herds during HPAI-H7N7 epizootic in 2003 in the Netherlands [37]. Moreover, detection of antibodies directed against human H3 in pig #2 would suggest that IAV transmission from human to pig also occurred within the farm. This is supported by the fact that 2016–2017 seasonal epidemic was mainly due to H3N2 in France [38], but indications that the farmer or other people in contact with the backyard pigs had been infected in December 2016 are lacking.

In mixed-herd #9, no HI titer was obtained for any of 22 ELISA-positive sera from batch A using the swine (except H1_hu_N2_Δ146–147_), avian and human antigens listed in Table 1. Additional analyses were implemented for ten selected sera with eleven other LPAI (H1-H6; H8-H9; H11) representative of strains that were previously detected in the concerned region, but they all gave negative results (data not shown). Thus, the infecting IAV was not identified in this study despite the use of a large panel of antigens. However, the presence of anti-IAV antibodies in sera from herd #9 (batch A) was confirmed by analyses using three other ELISA kits (ID Screen^®^ Influenza A Nucleoprotein Swine Indirect, IDVet, Grabels, France; PrioCHECK™ Swine Influenza Ab Serum Plate Kit, Thermo Fisher Scientific, Lelystad, The Netherlands; INgezim Influenza Porcina, Eurofins, Madrid, Spain) (data not shown). It is hypothesized that these pigs were most probably infected before arriving in herd #9, possibly with a swIAV strain antigenically distant from the reference strains used in this study, as uncommon strains were previously detected sporadically in this region [14].

## 4. Conclusions

In this study, we provided serological evidence that one backyard pig would have been infected naturally after close contact with HPAI-H5N8 clade 2.3.4.4b contaminated domestic ducks. The panel of antigens included in the HI tests, the cross-HI tests performed with specific HIS produced in SPF pigs, the successive samplings over time and the different results obtained in the ten investigated herds support the results, avoiding any misinterpretation that would have been related to a non-specific reaction. This is in accordance with a challenge study that demonstrated pigs are susceptible to infection with an HPAI-H5N8 Gs/Gd-lineage, clade 2.3.4.4 from North America, although with subclinical signs and a low humoral immune response [18]. Whereas the investigations conducted in our study do not indicate that HPAI H5N8 Gs/Gd-lineage clade 2.3.4.4b virus poses a high risk for pig health in contaminated mixed herds, the detection of a seropositive pig raises questions about the virus ability to cross the mammalian species barrier. In 2014, a dog was detected seropositive for HPAI-H5N8 virus in a contaminated poultry farm in Korea, which also illustrated virus transmission to mammals. The ability of dogs to be infected without clinical signs and to further transmit the HPAI-H5N8 virus, while at a low extent, was demonstrated experimentally [39]. In silico analyses, in vitro studies and experimental infections in the ferret model indicated that the risk HPAI-H5N8 infects mammals (including humans) is much weaker than the risk evaluated for HPAI-H5N1, HPAI-H5N6 or HPAI-H7N7 [3,7,40,41,42]. However, this study confirms caution should be paid to pigs housed in mixed farms where poultry is HPAI-H5N8 infected. From a physiological perspective, pigs are no more susceptible to avian IAV infection than humans, but some breeding practices and environments could favor virus transmission, leading pigs to contribute to adaptation of avian IAV to the mammalian host [43]. Even if knowledge of epidemiological risk factors for avian IAV infections in pigs must still be improved [44], it is well known that pigs are mixing vessels for the generation of new reassortant IAVs thanks to co-infections [9,45]. Such a reassortment event involving an avian H5 virus and a human-like H3N2 swIAV was reported in a pig in China in 2008 [46]. New reassortant viruses may exhibit gene constellations that could promote infectivity and transmission in swine, as shown experimentally for an HPAI-derived H5N1 virus that acquired H1N1pdm internal genes [47]. Moreover, ongoing evolution of HPAI-H5Nx clade 2.3.4.4b viruses may enable them to increase their capacity to transmit and adapt to mammals [48]. No human case of infection was identified in Europe during HPAI-H5N8 outbreaks in 2014/2015 and 2016/2017 [42,49], but Russia has recently reported to the World Health Organization the first case of a A(H5N8) being passed to workers exposed to contaminated bird flocks in December 2020 [50].

Altogether, these data indicate that continued surveillance of IAVs in pigs is warranted, including in areas of domestic poultry outbreaks. They recall that appropriate protective and biosecurity measures must be implemented in pig and poultry farms, integrating airborne transmission [51] and virus persistence in the environment [52]. Such measures should allow avoiding, as far as possible, IAV interspecies transmission, a prerequisite for co-infections and subsequent emergence of new viral genotypes whose impact on both animal and human health cannot be predicted. A One Health approach, involving swine, poultry and human health sectors, is undoubtedly a key to prevent the emergence and spread of new IAVs in mammals.

## Figures and Tables

**Table 1:** Characteristics of visited pig herds, related avian influenza outbreaks, sampled pigs and results of serological analyses towards influenza A virus infection by ELISA.

Herd		Avian Influenza Outbreak		Sampled Pigs		ELISA Results ^1^		
ID	Département of Location	HPAI-H5Nx Confirmed Case (Date)	Affected Poultry Species	Type of Pig Breeding	Herd Size	Physiological Stage or Age	Sampling Date (m/d/y)	No. Positive/No. Tested
#1	Dordogne	NA	NA	outdoor farrowing-to-finishing	134 sows, 500 post-weaning, 2000 finishing	sows	02/01/2017	10/10
#2	Tarn	H5N8 (12/1/2016)	ducks	outdoor finishing (backyard)	2	growing pigs	12/23/2016	1/2
							02/01/2017	2/2
							05/31/2017	1/1
#3	Tarn	H5N8 (12/2/2016)	ducks, geese	finishing	185	12 weeks	12/27/2016	0/28
						16 weeks	01/27/2017	0/15
#4	Gers	H5Nx (12/22/2016)	nd	finishing	nd	1 year	01/02/2017	0/1
							01/27/2017	0/1
#5	Gers	H5N8 (12/1/2016)	nd	nd	nd	nd	01/16/2017	0/2
#6	Gers	H5N8 (12/22/2016)	ducks	nd	nd	growing pigs	12/29/2016	0/5
#7	Pyrénées Atlantiques	H5Nx (nd)	nd	nd	nd	12 weeks	02/02/2017	0/60
#8	Hautes Pyrénées	H5N8 (12/26/2016)	ducks	post-weaning	nd	7 weeks	12/29/2016	0/60
#9	Landes	H5N8 (2/13/2017)	ducks, geese	outdoor post weaning-finishing	nd	10 weeks	05/09/2017	13/30(A)
						13 weeks	05/29/2017	9/30(A)
						17 weeks	05/09/2017	0/30(B)
						20 weeks	05/29/2017	0/30(B)
#10	Landes	H5N8 (12/27/2016)	ducks, geese	post weaning-finishing	nd	24 weeks	05/12/2017	0/60

^1^ A = first batch of pigs; B = second batch of pigs.; ID: identifier; No.: number; NA: not applicable; nd: not determined.

**Table 2:** Cross-hemagglutination inhibition (HI) assays between hyper-immune sera produced in SPF pigs inoculated with swine or avian influenza A virus strains, and reference swine, avian and human antigens.

Antigens ^1^		HI Titers of Swine Hyperimmune Sera Containing Antibodies Directed Against								
		Swine Influenza A Virus ^1^						Avian Influenza A Virus ^1^		
	HA-Clade [34,35]	1C.2.1	1C.2	1A.3.3.2	1B.1	1B.1.2.3	H3	H5-2.3.4.4	H5-Others	
	Subtype	H1_av_N1	H1_av_N2	H1N1pdm	H1_hu_N2	H1_hu_N2_Δ146–147_	H3N2	H5N8	H5N9	H5N3
**Swine influenza A virus**										
A/sw/Cotes d’Armor/0388/2009 (H1_av_N1)		**640**	**20**	<10	<10	<10	<10	<10 ^£^	<10 ^£^	<10 ^£^
A/sw/France/65-150242/2015 (H1_av_N2)		**80 ^£^**	**1280 ^£^**	<10 ^£^	<10 ^£^	<10 ^£^	<10 ^£^	nd	nd	nd
A/sw/Sarthe/0255/2010 (H1N1pdm)		10	nd	**640**	10	<10	<10	nd	nd	nd
A/sw/France/57-140136/2014 (H1N1pdm)		**20**	<10 ^£^	**640**	**20**	<10	<10	<10 ^£^	<10 ^£^	<10 ^£^
A/sw/Scotland/410440/1994 (H1_hu_N2)		<10	<10 ^£^	10	**1280**	**40**	10	<10 ^£^	<10 ^£^	<10 ^£^
A/sw/France/22-130212/2013 (H1_hu_N2_Δ146–147_)		10	10 ^£^	<10	**80**	**1280**	10	<10 ^£^	<10 ^£^	<10 ^£^
A/sw/Flandres/1/1998 (H3N2)		<10	10 ^£^	<10	<10	<10	**2560**	<10 ^£^	<10 ^£^	<10 ^£^
**Avian influenza A virus**										
A/decoyduck/France/161105a/2016 (H5N8)		<10 ^£^	nd	<10 ^£^	<10 ^£^	<10 ^£^	<10 ^£^	**50**	<10	<10
A/muteswan/Croatia/102/2016 (H5N5)		nd	nd	nd	nd	nd	nd	**20 ^£^**	<10 ^£^	<10 ^£^
A/duck/France/150236b/2015 (H5N9)		10 ^£^	nd	<10 ^£^	<10 ^£^	<10 ^£^	<10 ^£^	<10	**25**	**120**
A/muscovyduck/France/070090b/2007 (H5N3)		<10 ^£^	nd	<10 ^£^	<10 ^£^	<10 ^£^	<10 ^£^	<10	10	**160**
A/mallard/France/100204f/2010 (H3N8)		nd	nd	nd	nd	nd	nd	<10 ^£^	<10 ^£^	<10 ^£^
A/pekinduck/France/090173/2009 (H6N8)		nd	nd	nd	nd	nd	nd	<10 ^£^	<10 ^£^	<10 ^£^
**Human influenza A virus**										
A/Victoria/361/2011 (H3N2)		<10 ^£^	nd	<10 ^£^	10 ^£^	nd	<10 ^£^	<10 ^£^	<10 ^£^	<10 ^£^

The table reports mean HI titers obtained from four independent assays or from one assay when character £ is indicated. Homologous HI titers are underlined. HI titers equal or above the positive threshold (20) are indicated in bold. ^1^ Swine and avian influenza A virus strains used to produce hyperimmune sera were those included as antigens in the cross-HI tests. Anti-H1N1pdm HIS was produced against A/sw/Sarthe/0255/2010. nd: not determined; sw = swine.

**Table 3:** Hemagglutination inhibition (HI) titers obtained for individual sera taken between December 2016 and May 2017 in non-mixed herd #1 (ten sows) and mixed herd #2 (two backyard pigs).

Herd ID	Sampling Date	Pig ID	ELISA S/P ^2^		HI Titres Obtained with Influenza A Virus Strains ^1^ of Different Origins, Subtypes and Lineages											
					Swine IAV					Avian IAV						Human IAV
				HA Clade	1C.2.1	1A.3.3.2	1B.1	1B.1.2.3	H3	H5-2.3.4.4		H5-Others		Hx(N8)		H3
				Subtype	H1_av_N1	H1N1pdm	H1_hu_N2	H1_hu_N2 Δ146–147	H3N2	H5N8	H5N5	H5N9	H5N3	H3N8	H6N8	H3N2
#1	01 Feb	#1	**21.8**		10	<10	<10	<10	<10	<10	nd	<10	<10	nd	nd	nd
		#2	**4.7**		**80**	**80**	<10	<10	<10	<10	nd	<10	<10	nd	nd	nd
		#3	**13.0**		**20**	<10	<10	<10	<10	<10	nd	<10	<10	nd	nd	nd
		#4	**18.8**		**20**	**40**	<10	<10	<10	<10	nd	<10	<10	nd	nd	nd
		#5	**22.3**		<10	<10	<10	<10	<10	<10	nd	<10	<10	nd	nd	nd
		#6	**5.1**		**20**	**20**	<10	<10	<10	<10	nd	<10	<10	nd	nd	nd
		#7	**5.9**		**40**	**80**	<10	<10	<10	<10	nd	<10	<10	nd	nd	nd
		#8	**18.2**		<10	**20**	<10	<10	<10	<10	nd	<10	<10	nd	nd	nd
		#9	**5.4**		**40**	**160**	<10	<10	<10	<10	nd	<10	<10	nd	nd	nd
		#10	**5.4**		10	10	<10	<10	<10	<10	nd	<10	<10	nd	nd	nd
#2	23 Dec	#1	**10.2**		<10	<10	<10	<10	<10	**40**	**20**	<10	<10	<10	<10	10
	23 Dec	#2	58.5		<10	<10	<10	<10	<10	<10	<10	<10	<10	<10	<10	**20**
	01 Feb	#1	**24.8**		<10	<10	<10	<10	<10	**40**	**20**	<10	<10	<10	<10	<10
	01 Feb	#2	**9.1**		<10	<10	<10	<10	<10	<10	<10	<10	<10	<10	<10	**20**
	31 May	nd	**41.7**		<10	<10	<10	nd	<10	<10	nd	<10	<10	nd	nd	10

Results above the test positive threshold (20) are indicated in bold. Results obtained in ELISA are indicated as S/P values for information. ^1^ Strains used as antigens are given in the main text and in Table 2. ^2^ S/P is result of competition ELISA obtained by the following calculation = OD sample/OD negative control*100. If the result is ≤45%, the sample is interpreted positive; comprised between 45 and 50% it is doubtful; ≥50% it is negative. ID: identifier; nd: not determined.

## Data Availability

Individual and/or raw data obtained in this study are available on request from the corresponding author.
